# CRISPR/Cas9-mediated genome editing of *RsGL1a* and *RsGL1b* in radish (*Raphanus sativus* L.)

**DOI:** 10.3389/fpls.2022.951660

**Published:** 2022-10-13

**Authors:** Naoki Muto, Takashi Matsumoto

**Affiliations:** Department of Bioscience, Faculty of Life Sciences, Tokyo University of Agriculture, Tokyo, Japan

**Keywords:** CRISPR/Cas9, single guide RNA (sgRNA), gene editing, *Raphanus sativus* L., *RsGL1a*, *RsGL1b*, trichome, molecular breeding

## Abstract

The clustered regularly interspaced short palindromic repeats (CRISPR)/CRISPR-associated protein 9 (Cas9) is a powerful tool widely used for genome editing in various organisms, including plants. It introduces and facilitates the study of rare genetic mutations in a short time and is a potent tool to assist in plant molecular breeding. Radish (*Raphanus sativus* L.) is an important Brassicaceae vegetable cultivated and consumed worldwide. However, the application of the CRISPR/Cas9 system is limited by the absence of an efficient transformation system in radish. This study aimed to establish a CRISPR/Cas9 system in radish employing the *Agrobacterium*-mediated genetic transformation system reported recently. For this purpose, we performed genome editing using the CRISPR/Cas9 system targeting the *GLABRA1* (*GL1*) orthologs, *RsGL1a* and *RsGL1b*, that induces leaf trichome formation in radish. A Cas9/single guide RNA (sgRNA) vector with a common sgRNA corresponding to *RsGL1a* and *RsGL1b* was transferred. A total of eight T_0_ plants were analyzed, of which six (editing efficiency 75%) had a mutated *RsGL1a*, five (62.5%) had a mutated *RsGL1b*, and five showed mutations in both *RsGL1a* and *RsGL1b*. Most mutations in T_0_ plants were short (<3 bp) deletions or insertions, causing frameshift mutations that might produce non-functional proteins. Chimeric mutations were detected in several T_0_ generation plants. In the T_1_ generation, the hairless phenotype was observed only in plants with knockout mutations in both *RsGL1a* and *RsGL1b*. The majority of mutant alleles in T_0_ plants, with the exception of the chimeric mutant plants detected, were stably inherited in the T_1_ generation. In conclusion, we successfully knocked out *RsGL1a* and *RsGL1b* using the CRISPR/Cas9 system and demonstrated that both *RsGL1a* and *RsGL1b* independently contribute to the induction of leaf trichome formation in radish. In this study, genome-edited plants without T-DNA, which are useful as breeding material, were obtained. The findings prove the feasibility of genome editing in radish using a CRISPR/Cas9 system that could accelerate its molecular breeding to improve agronomically desirable traits.

## Introduction

Radish (*Raphanus sativus* L.), characterized by enlarged hypocotyls and taproots, belongs to the Brassicaceae family and is an important vegetable cultivated and consumed worldwide. The edible parts of radish including the roots, leaves, and sprouts, are of high nutritional value (Hara et al., 2011; Banihani, 2017). Therefore, the genetics and genomics of radish have been the subject of extensive research interest. Rapid advances in sequencing technologies have enabled the sequencing of whole genomes of five cultivated radish varieties and the development of an open-source genome database (Kitashiba et al., 2014; Mitsui et al., 2015; Xiaohui et al., 2015; Jeong et al., 2016; Shirasawa et al., 2020), which facilitates the genetic researches in radish. Mitsui et al. (2015) showed that the *R. sativus* originates from a divergence of the *Brassica rapa* and has a triplicated genome compared to *Arabidopsis thaliana*. Given its close relatedness with other *Brassica* crops (Chen et al., 2011) and *A. thaliana*, it is expected that the availability of genetic information and experimental techniques from these crops could be extended to the development of molecular breeding and gene function analysis in radish.

The clustered regularly interspaced short palindromic repeats (CRISPR)/CRISPR-associated protein 9 (Cas9) system, derived from the immune system of bacteria and archaea (Gasiunas et al., 2012; Jinek et al., 2012), is composed of the Cas9 endonuclease and a synthetic single-guide RNA (sgRNA), which directs the Cas9 protein to the targeted genomic DNA (gDNA) sequence preceding the protospacer-associated motif (PAM). CRISPR/Cas9 has gained tremendous popularity for targeted mutagenesis in transformable eukaryotic species (Cardi et al., 2017). CRISPR/Cas9 recognizes and cleaves the 20-mer target sequence and the 5’-NGG-3’ (PAM) sequence and frequently introduces one or two base insertions or deletions (in/del) during the repair of these cleavages, resulting in frameshift mutations to produce gene knockouts (Ran et al., 2013). Genome editing with CRISPR/Cas9 has been widely applied to many plant species (Cardi et al., 2017; Mohd Saad et al., 2021; Tian et al, 2021; Liu et al., 2022). Furthermore, it has also been used to assess the gene function in many plant species owing to its simplicity and easy accessibility. However, in radish, although several gene functions and their effects on traits, such as root color (Lai et al., 2020) and root shape (Gancheva et al., 2016), have been speculated, most of the functions of the genes have not been validated because of the difficulty of producing gene knockouts. Moreover, genome editing by CRISPR/Cas9 in radish was limited by the unavailability of an efficient transformation system and the difficulties to introduce the CRISPR/Cas9 gene and sgRNA. Recently, we have successfully established a highly efficient method for *Agrobacterium*-mediated genetic transformation in radish (Muto et al., 2021). The method enabled the successful and efficient transformation of radish hypocotyl explants, suggesting its potential to accelerate genome editing and gene function analysis in radish.

The leaf hairs (trichomes) extend from the epidermis of aerial tissues of plants and serve as a physical barrier against biotic (Kariyat et al., 2018) and abiotic (Wu and Kao, 2009; Bickford, 2016; Escobar-Bravo et al., 2018) stress. In *A. thaliana*, the leaf trichome has been explored as an excellent model system to study plant differentiation at a single-cell level (Kryvych et al., 2008; Kryvych et al., 2011). Furthermore, trichomes can be visually assessed, and the presence/absence of the trichomes can be used to measure gene knockout efficiency, making it a suitable model to investigate the efficiency of genome editing (Stuttmann et al., 2021). It has also been reported that trichomes play an important role in imparting textures to vegetables (Kawakatsu et al., 2017); therefore, understanding the genetic mechanism of trichome development in radish can lead to the breeding of radishes with suitable edible leaves. *GLABRA1* (*GL1*), a member of the R2R3 MYB transcription factor family (Oppenheimer et al., 1991), is known to control the presence of leaf hair in Brassicaceae plants (Bloomer et al., 2012; Li et al., 2013; Kawakatsu et al., 2017). *GL1* and the bHLH transcription factor *GLABRA3* (*GL3*) form a transcriptional activator complex that regulates the transcription of *GLABRA2* (*GL2*) and induces leaf hair formation (Wang and Chen, 2008). In radish, two *GL1* orthologs—*RsGL1a* and *RsGL1b*—have been predicted to induce leaf hair formation (Li et al., 2013). Therefore, we hypothesized that using these two genes as targets could provide insights into the efficiency of the CRISPR/Cas9 system in radish.

In this study, we aimed to explore the potential of the CRISPR/Cas9 system in radish. For this purpose, we performed the CRISPR/Cas9-mediated genome editing of *RsGL1a* and *RsGL1b*. To the best of our knowledge, this study, for the first time, demonstrates the knockout of *RsGL1a* and *RsGL1b* using the CRISPR/Cas9 system targeting the two genes simultaneously. It shows that the knockout genes obtained in the T_0_ generation were inherited and displayed the desired phenotype in the T_1_ generation. These results provide evidence for the potential of the CRISPR/Cas9 system to accelerate molecular breeding and gene function analysis in radish.

## Materials and methods

### Target sequences selection and sgRNA design

**Figure 1:**
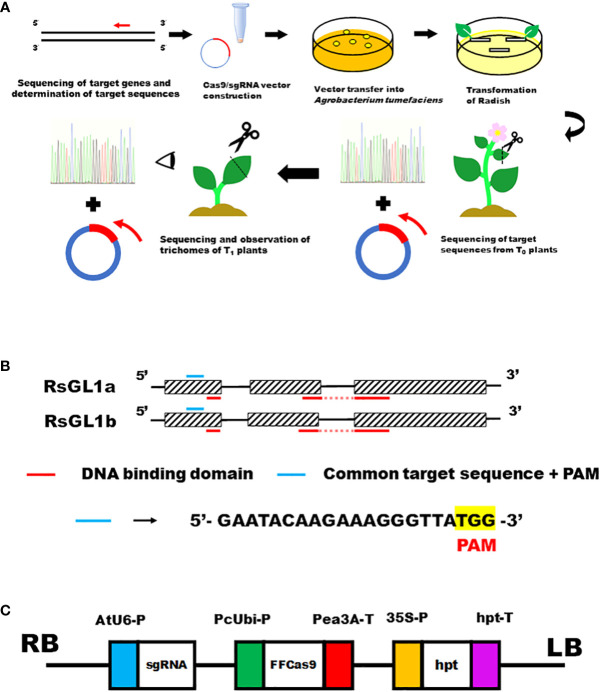
CRISPR/Cas9-mediated genome editing targeting leaf trichome formation genes *RsGL1a* and *RsGL1b* in radish. **(A)** The workflow of transformation and genome editing in radish. **(B)** Schematic presentation of the domain structures and location of the target sequences of *RsGL1a* and *RsGL1b*. Figures consisting of boxes and lines show *RsGL1a* and *RsGL1b* genes. Boxes indicate exons, lines indicate introns, blue lines indicate common target sequences + PAM sequence, and red lines indicate DNA binding domains. **(C)** The schematic view of the constructed pZH_gGL1_Cas9 vector. AtU6-P, *Arabidopsis thaliana* Ubiquitin 6 gene promoter; sgRNA, single guide RNA; PcUbi-P, *Petroselinum crispum* Ubiquitin gene promoter; FFCas9, Cas9 gene used by Fauser et al., 2014; Pea3A-T, pea RBCS3A (pea3A) terminator from *Pisum sativum*; 35S-P, cauliflower mosaic virus (CaMV) 35S promoter; *hpt*, hygromycin phosphotransferase gene; hpt-T, hpt terminator.

The workflow of transformation and genome editing in radish is shown in **Figure 1A**. gDNA of the radish cultivar ‘Pirabikku’ (*R. sativus* L. var. sativus) was extracted using the NucleoSpin Plant II kit (MACHEREY-NAGEL GmbH & Co. KG, Germany). Sequence information for *RsGL1a* (Accession Number AB747346.1) and *RsGL1b* (Accession Number AB747347.1) were obtained from NCBI (https://www.ncbi.nlm.nih.gov/) to design PCR primers (**Supplementary Table 1**).To amplify *RsGL1a* and *RsGL1b*, PCR was performed using Blend Taq (TOYOBO, Japan) and the following PCR conditions: 95°C for 2 min, followed by 35 cycles of 30 s at 95°C, 30 s at 60°C, and 30 s at 72°C. The PCR products obtained were cloned into the TArget Clone vector (TOYOBO, Japan). The vector and PCR products were ligated using the DNA Ligation Kit Ver.1 (TaKaRa, Japan). The ligated plasmid was transferred into *Escherichia coli* DH5α cells, amplified, and purified using QIAprep Spin Miniprep Kit (QIAGEN, Netherlands). To determine the nucleotide sequences of *RsGL1a* and *RsGL1b*, the purified plasmid was Sanger sequenced using the universal primer M13 (5’-GAGCGGATAACAATTTCACACAGG -3’). The protein sequences of RsGL1a and RsGL1b were predicted, and protein domain analysis was performed using PROSITE (https://prosite.expasy.org/) (**Figure 1B**).

### Vector construction

A binary vector was constructed using restriction enzymes and ligation following the method described in the previous studies (Ito et al., 2015; Mikami et al., 2015; Nakajima et al., 2017). The single guide RNA (sgRNA) cloning vector (pUC19_AtU6_ccdB_oligo) and the binary vector (pZH_gYSA_FFCas9) were obtained from Dr. Makasi Endo from The National Agriculture and Food Research Organization (Tsukuba, Japan). In pZH_gYSA_FFCas9A, the *neomycin phosphotransferase II* (*nptII*) cassette of the vector originally described in Ito et al. was replaced with a *hygromycin phosphotransferase* (*hpt*) cassette (Mikami et al., 2015). The common target sequence of *RsGL1a* and *RsGL1b*: 18-mer + BbsI site (5’- ATTG GAATACAAGAAAGGGTTA -3’) and sthe complementary oligo + BbsI site (5’- AAAC TAACCCTTTCTTGTATTC -3’) were incubated at 95°C for 5 min, and then at 25°C for 20 min for annealing. The annealed oligos were ligated to the BbsI site of the sgRNA-scaffold of the cloning vector (pUC19_AtU6_ccdB_oligo) and the ligated plasmid was transferred into *E. coli* DH5α cells. Double screening (100mg/L ampicillin selection and survival selection) detected clones in which ccdB gene was replaced with the GL1 target sequence. For the screened candidates, sequencing was performed using the AtU6 primer nearby the target sequence (5’- TGTTTATACAGCTTACATTTTCTTGAACCGTAGCT -3’) to confirm the 18-mer insert. The constructed sgRNA vector and the binary vector (pZH_gYSA_FFCas9) with the Cas9 gene and *hygromycin phosphotransferase* (*hpt*) were digested with I*-*SceI. Then, the sgRNA cassette was ligated into the binary vector and transferred into *E. coli* DH5α, selected with 100 mg/L streptomycin. The positive colonies were cultured, and plasmid DNAs were purified. The completed pZH_gGL1_Cas9 vector was digested with EcoRI to confirm that the sgRNA cassette was in the same direction as that of the Cas9 cassette. pZH_gGL1_Cas9 vector (**Figure 1C**) was then transferred into *Agrobacterium tumefaciens* strain GV3101 *via* electroporation (Mersereau et al., 1990). Although the 35S promoter from Cauliflower Mosaic Virus is used for plant-specific gene expression, it also works in bacteria including *Agrobacterium* (Jacob et al., 2002; Muto et al., 2021). The plasmid-transformed *Agrobacterium* was cultured in YEP medium containing 50 mg/L hygromycin on the agar plate, and a few colonies were cultured in the liquid YEP medium containing 50 mg/L hygromycin and stored at −80°C as glycerol stock.

### Detection of plant transformation and restorer of fertility gene

The *Raphanus* transformation was carried out following the procedure described in our previous study (Muto et al., 2021). Transformed plants were selected with 10 mg/L hygromycin. To verify the introduction of T-DNA, gDNA of the regenerated plants was extracted, and PCR was performed using Hpt_F and Hpt_R primers (**Supplementary Table 1**) derived from the sequence of *hpt* using the Quick Taq HS DyeMix (TOYOBO, Japan). The PCR conditions were as follows: 95°C for 2 min, followed by 25 cycles of 30 s at 95°C, 30 s at 60°C, and 30 s at 72°C. The mitochondrial male sterility gene, *orf138*, and the nuclear restorer of fertility gene (Rf gene) *orf687* are present in radish (Yasumoto et al., 2008). The radish cv. ‘Pirabikku’ with Rf-type *orf687* produces pollen (Muto et al., 2021); therefore, we screened for Rf-type *orf687* in this study using the PCR-Restriction Fragment Length Polymorphism (RFLP) assay. The PCR was performed using the orf687_F and orf687*_*R primers (**Supplementary Table 1**), and the PCR products were digested with SspI. The T_0_ plants with T-DNA containing the Cas9 gene and Rf-type *orf687* were used for further experiments. The flowering and pollen formation in these selected transgenic plants were confirmed to ensure fertility.

### Confirmation of CRISPR/Cas9-mediated mutation in T_0_ plants

A total of eight transgenic T_0_ plants were analyzed to confirm the CRISPR/Cas9-mediated mutations. Reportedly, in *Brassica napus*, the mutant gene from the regenerating plants are maintained in only a small portion of the mature plants (Okuzaki et al., 2018). To confirm the mutation of *RsGL1a* and *RsGL1b* in mature plants, we extracted gDNA from the upper leaf of the flowering plant and performed PCR using RsGL1a_PCR_F, RsGL1a_PCR_R, RsGL1b_PCR_F, and RsGL1b_PCR_R primers (**Supplementary Table 1**). The PCR conditions were as follows: 95°C for 2 min, followed by 35 cycles of 30 s at 95°C, 30 s at 60°C, and 30 s at 72°C. The PCR products were then used for direct sequencing using the RsGL1a_seq and RsGL1b_seq primers (**Supplementary Table 1**). Chromatograms and nucleotide sequences (text data) were obtained. The chromatograms with a single peak and the nucleotide sequence identical to the wild-type allele were defined as “Wild-type” (no mutation). The multi-peaks near the target sequence in the chromatograms were defined as “Heterogeneous”. The chromatograms with a single peak and a nucleotide sequence other than the wild-type allele were defined as “Homogeneous” (**Table 1**). The exact allele sequences of the five plants with mutations in both *RsGL1a* and *RsGL1b* were obtained. The PCR products of the *RsGL1a* and *RsGL1b* were cloned into DH5α and sequenced according to the method previously described. The nucleotide sequences were aligned using NCBI BLAST (**Table 2**). Subsequently, the self-pollinated seeds of plants with mutations in both *RsGL1a* and *RsGL1b* were collected.

**Table 1:** Detection of mutations in *RsGL1a* and *RsGL1b* of T_0_ generation plants.

T_0_ plant No.	Mutation types		Double mutations
	*RsGL1a*	*RsGL1b*	
#1	Wild type	Wild type	
#2	Heterogeneous	Heterogeneous	Yes
#3	Homogeneous	Heterogeneous	Yes
#4	Heterogeneous	Heterogeneous	Yes
#5	Heterogeneous	Heterogeneous	Yes
#6	Homogeneous	Heterogeneous	Yes
#7	Heterogeneous	Wild type	
#8	Wild type	Wild type	
Editing efficiency	6/8 (75%)	5/8 (62.5%)	5/8 (62.5%)

**Table 2:** Alignment analysis of *RsGL1a* and *RsGL1b* in T_0_ generation plants.

T_0_ plant No.	Mutation	*RsGL1a*	Clones/total	Mutation	*RsGL1b*	Clones/total
	WT	AGGAATACAAGAAAGGGTTATGGACA		WT	AAGAATACAAGAAAGGGTTATGGACA	
#2	AT in	AGGAATACAAGAAAGGGATTTATGGACA	7/8	T in	AAGAATACAAGAAAGGGTTTATGGACA	7/8
	3 del	AGGAATACAAGAAAGGG—TGGACA	1/8	C in	AAGAATACAAGAAAGGGCTTATGGACA	1/8
#3	T in	AGGAATACAAGAAAGGGTTTATGGACA	6/8	WT	AAGAATACAAGAAAGGGTTATGGACA	2/8
	A in	AGGAATACAAGAAAGGGATTATGGACA	1/8	A in	AAGAATACAAGAAAGGGATTATGGACA	4/8
	5 del	AGGAATACAAGAAAGGG—–GACA	1/8	T in	AAGAATACAAGAAAGGGTTTATGGACA	1/8
				C in	AAGAATACAAGAAAGGGCTTATGGACA	1/8
				5 in	AAGAATACAAGAAAGGGAAGGGTTATGGACA	1/8
				T sub	AAGAATACAAGAAAGG**T**TTATGGACA	1/8
#4	A in	AGGAATACAAGAAAGGGATTATGGACA	5/8	WT	AAGAATACAAGAAAGGGTTATGGACA	6/8
	GG del	AGGAATACAAGAAAG–TTATGGACA	3/8	G del	AAGAATACAAGAAAGG-TTATGGACA	2/8
#5	TT del	AGGAATACAAGAAAGGG–ATGGACA	4/8	A in	AAGAATACAAGAAAGGGATTATGGACA	4/8
	2 del, 1 sub	AGGAATACGAGAAA**A**–TTATGGACA	3/8	18 del, 22 in	<—————>TTATGGACA	4/8
	T in	AGGAATACAAGAAAGGGTTTATGGACA	1/8			
#6	A in	AGGAATACAAGAAAGGGATTATGGACA	8/8	WT	AAGAATACAAGAAAGGGTTATGGACA	1/8
				T in	AAGAATACAAGAAAGGGTTTATGGACA	6/8
				70 del	<—————>TTATGGACA	1/8

WT, Wild Type; in, insertion; del, deletion; sub, substitution. PAM sequences are underlined. Insertions are shown in red font. Deletions are shown using hyphens. Substitutions are shown in bold font. In/del greater than 3-bp are shown using angle brackets and hyphens.

### Off-target analysis

The sgRNA (5’-GAATACAAGAAAGGGTTA-3’) corresponding to *RsGL1a* and *RsGL1b* were analyzed using CRISPR RGEN Tools (http://www.rgenome.net/cas-offinder/). Off-target analysis was performed on *Raphanus sativus* (Rs1.0, genome assembly from cultivar WK10039) as a reference, and a gene with a single nucleotide mismatch (5’-GAATACAAGAAAGGTTTATGG-3’) was detected. This gene was a *Raphanus* ortholog gene of *Arabidopsis AtMYB23* (Kirik et al., 2001; Kirik et al., 2005). To evaluate the possibility of this gene being modified with the CRISPR/Cas9, primers were designed to amplify the region around an off-target sequence of this *RsMYB23*-like gene (RsMYB23_F and RsMYB23_R; **Supplementary Table 1**). The PCR products of the eight transgenic T_0_ plants were obtained. Nucleotide sequences were obtained from the PCR products by direct sequencing to confirm the mutations using sequence primer (RsMYB23_SEQ; **Supplementary Table 1**).

### Confirmation of CRISPR/Cas9-mediated mutation in T_1_ plants

Leaf trichomes of T_1_ plants were visually observed, and the number of hairy and hairless plants was counted. (**Table 3**). Two progenies with hairy and hairless phenotypes were randomly selected at T_1_ generation, and gDNA was extracted from each progeny. The PCR of the target region was performed as in the T_0_ analysis. Homogeneous/Heterogeneous mutation was detected in the chromatograms with direct Sanger sequencing of the PCR products of either *RsGL1a* and *RsGL1b*, and inheritance of the mutations observed at T_0_ generation to T_1_ generation was verified by the basecalls. If there were multiple peaks near the target sequence, each DNA molecule was cloned into *E.coli* and an individual sequence was identified. Furthermore, as T_1_ plants are considered to be anchored to two genes (*RsGL1a* and *RsGL1b*), a minimum of four clones were analyzed to detect alleles from both genes (**Tables 4A**, **B**).

**Table 3:** Observation of trichome formation in T_1_ generation plants.

T_0_ plant No. originated	Hairy	Hairless	Total
#3	17	2	19
#4	10	4	14
#5	0	17	17
#6	19	4	23

**Table 4A:** Alignment analysis of *RsGL1a* in hairless and hairy phenotypes of T_1_ generation plants.

T_1_ plant No.	Phenotype	Mutation	*RsGL1a*	Clones/Total	Detected in T_0_	T-DNA
		WT	AGGAATACAAGAAAGGGTTATGGACA			
#3-4	Hairless	14 del	AGGAATACAA————–CA	3/5	No	+
		33 del	AGGAATACAAG<————>^a^	2/5	No	
#3-16	Hairless	7 del	AGGAATACAAGACGAC——ACA	3/4	No	+
		T del	AGGAATACAAGAAAGG-TTATGGACA	1/4	No	
#3-3	Hairy	WT	AGGAATACAAGAAAGGGTTATGGACA	1/7	No	+
		T in	AGGAATACAAGAAAGGGTTTATGGACA	4/7	Yes	
		G in	AGGAATACAAGAAAGGGGTTATGGACA	1/7	No	
		2 del, 32 in	AGGAATACAAGAAAG<>TTATGGACA	1/7	No	
#3-18	Hairy	A in	AGGAATACAAGAAAGGGATTATGGACA	–	Yes	+
#4-6	Hairless	A in	AGGAATACAAGAAAGGGATTATGGACA	–	Yes	–
#4-7	Hairless	A in	AGGAATACAAGAAAGGGATTATGGACA	3/4	Yes	+
		TT del	AGGAATACAAGAAAGGG–ATGGACA	1/4	No	
#4-4	Hairy	A in	AGGAATACAAGAAAGGGATTATGGACA	3/4	Yes	–
		GG del	AGGAATACAAGAAAG–TTATGGACA	1/4	Yes	
#4-9	Hairy	A in	AGGAATACAAGAAAGGGATTATGGACA	–	Yes	+
#5-1	Hairless	TT del	AGGAATACAAGAAAGGG–ATGGACA	3/4	Yes	+
		2 del, 1 sub	AGGAATACGAGAAA**A**–TTATGGACA	1/4	Yes	
#5-2	Hairless	2 del, 1 sub	AGGAATACGAGAAA**A**–TTATGGACA	3/5	Yes	+
		A in	AGGAATACAAGAAAGGGATTATGGACA	2/5	Yes	
#6-1	Hairless	A in	AGGAATACAAGAAAGGGATTATGGACA	–	Yes	+
#6-2	Hairless	A in	AGGAATACAAGAAAGGGATTATGGACA	–	Yes	+
#6-3	Hairy	A in	AGGAATACAAGAAAGGGATTATGGACA	–	Yes	+
#6-4	Hairy	A in	AGGAATACAAGAAAGGGATTATGGACA	–	Yes	+

WT, Wild Type; in, insertion; del, deletion; sub, substitution. PAM sequences are underlined. Insertions are shown in red font. Deletions are shown using hyphens. Substitutions are shown in bold font. In/del greater than 3-bp are shown using angle brackets and hyphens. ^a^Splice mutation.

**Table 4B:** Alignment analysis of *RsGL1b* in hairless and hairy phenotypes of T_1_ generation plants.

T_1_ plant No.	Phenotype	Mutation	*RsGL1b*		Clones/Total	Detected in T_0_	T-DNA
		WT	AAGAATACAAGAAAGGGTTATGGACA				
#3-4	Hairless	T in	AAGAATACAAGAAAGGGTTTATGGACA		2/4	Yes	+
		T del	AAGAATACAAGAAAGGG-TATGGACA		2/4	No	
#3-16	Hairless	T in	AAGAATACAAGAAAGGGTTTATGGACA		-	Yes	+
#3-3	Hairy	WT	AAGAATACAAGAAAGGGTTATGGACA		3/4	Yes	+
		A in	AAGAATACAAGAAAGGGATTATGGACA		1/4	Yes	
#3-18	Hairy	WT	AAGAATACAAGAAAGGGTTATGGACA		2/4	Yes	+
		T in	AAGAATACAAGAAAGGGTTTATGGACA		1/4	Yes	
		TT del	AAGAATACAAGAAAGGG–ATGGACA		1/4	No	
#4-6	Hairless	G del	AAGAATACAAGAAAGG-TTATGGACA		-	Yes	-
#4-7	Hairless	G del	AAGAATACAAGAAAGG-TTATGGACA		-	Yes	+
#4-4	Hairy	WT	AAGAATACAAGAAAGGGTTATGGACA		3/6	Yes	-
		G del	AAGAATACAAGAAAGG-TTATGGACA		3/6	Yes	
#4-9	Hairy	WT	AAGAATACAAGAAAGGGTTATGGACA		1/4	Yes	+
		G del	AAGAATACAAGAAAGG-TTATGGACA		3/4	Yes	
#5-1	Hairless	A in	AAGAATACAAGAAAGGGATTATGGACA		3/6	Yes	+
		18 del, 22in	<—————>TTATGGACA		3/6	Yes	
#5-2	Hairless	A in	AAGAATACAAGAAAGGGATTATGGACA		-	Yes	+
#6-1	Hairless	T in	AAGAATACAAGAAAGGGTTTATGGACA		3/7	Yes	+
		14 del	AA————–GTTATGGACA		4/7	No	
#6-2	Hairless	T in	AAGAATACAAGAAAGGGTTTATGGACA		3/4	Yes	+
		14 del	AA————–GTTATGGACA		1/4	No	
#6-3	Hairy	T sub	AAGAATACAAGAAAGG**T**TTATGGACA		-	No	+
#6-4	Hairy	T sub	AAGAATACAAGAAAGG**T**TTATGGACA		-	No	+

WT, Wild Type; in, insertion; del, deletion; sub, substitution. PAM sequences are underlined. Insertions are shown in red font. Deletions are shown using hyphens. Substitutions are shown in bold font. In/del greater than 3-bp are shown using angle brackets and hyphens.

### Evaluation of trichome structure

**Figure 2:**
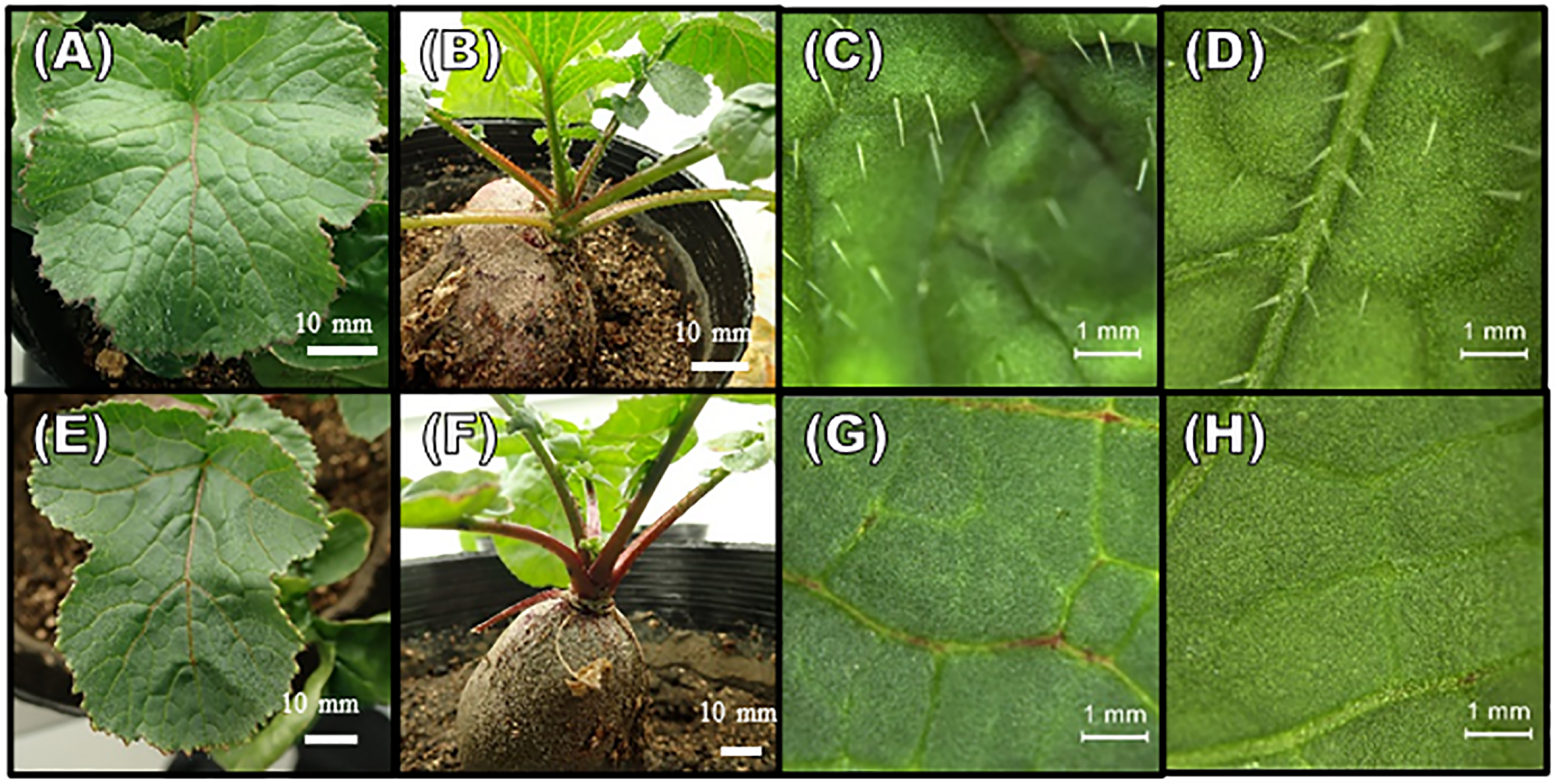
Phenotypes of trichome formation in T_1_ generation plants. **(A–D)** hairy plants; **(E–H)** hairless plants. **(A, E)** The adaxial surface of leaves, **(B, F)** petioles, and meristems. **(C, G)** Enlarged views of adaxial surface of leaves. **(D, H)** Enlarged views of abaxial surface of leaves. The scale bars in **(A, B, E, F)** are 10 mm, and in **(C, D, G, H)** 1 mm are shown.

The plants evaluated for trichomes were grown in a growth chamber, under the same conditions as those for the transformation, at 25°C under long-day conditions (16 h Light/8 h Dark) at a light intensity of 60 μmol m^-2^ s^-1^. The trichomes on the frontside and backside of the leaves of the T_1_ plants were observed visually. Leaves of plants with different densities of trichomes were photographed under a fluorescent stereomicroscope (Leica M165 FC) (**Figure 2**).

## Results

### Sequencing of target genes and determination of target sequences

The nucleotide sequences of the *RsGL1a* and *RsGL1b* are shown with the deduced protein sequences in **Supplementary Figure 1**. These gene sequences were submitted in DDBJ (https://www.ddbj.nig.ac.jp/index-e.html) for *RsGL1a* (Accession Number LC719984) and *RsGL1b* (Accession Number LC719985).

The identity of the **‘**Pirabikku**’** nucleotide sequences *RsGL1a* and *RsGL1b* with that of Ra38 DH line source, another *Raphanus* variety, was 1216/1307 (93%) and 1174/1311 (90%), respectively. The protein domain analysis identified two Myb DNA-binding domains for both *RsGL1a* and *RsGL1b*, suggesting the association of both genes in trichome formation as transcription factors. The 18-mer + PAM (5**’**-GAATACAAGAAAGGGTTA TGG -3**’**) sequence (21-mer) common to both genes located upstream of the Myb DNA-binding domain in the first exon of both genes were selected as target sequences (**Figure 1B**).

### Production of *RsGL1* edited T_0_ plants

In the eight T_0_ plants, the chromatograms of *RsGL1*a and *RsGL1b* sequences, obtained through direct sequencing, were analyzed (**Table 2**). Six of these plants had mutations in *RsGL1a* (editing efficiency 75%), two of which were homogeneous, whereas five plants had mutations in *RsGL1b* (editing efficiency 62.5%), and none were homogeneous. Double mutations were detected in five of the eight plants (editing efficiency 62.5%). The PCR products of *RsGL1a* and *RsGL1b* of the T_0_ plants with double mutations were cloned into *E. coli* and eight colonies from each PCR product were sequenced (**Table 2**). All mutations occurred at positions two or three bases upstream of the PAM sequence. Analysis of *RsGL1a* detected no wild-type alleles in any T_0_ plants. Most in/del were short (<3 bp) and caused frameshift mutations that might abolish protein function. The only deletion of 3 bp in plant #3 did not cause a frameshift mutation, indicating this deletion might not affect the gene function. We expected the following genotypes: homogenous (two alleles having the same mutation); heterogenous (only one allele is mutated); biallelic (two alleles having different mutations); chimeric (two or more different alleles are present in the sequence chromatogram), or wild-type. In T_0_ plants, the *RsGL1a* in #5, *RsGL1b* in #6, and both *RsGL1a* and *RsGL1b* in #3 were identified to have three or more different alleles, indicating that they have chimeric mutations. The *RsGL1a* of T_0_ plant #6 was identified to harbor a homogenous mutation both by direct sequencing and clone sequencing However, the *RsGL1a* of T_0_ plant #3, which was shown to have a homogeneous mutation by direct sequencing, had three different alleles. These cloning results were further confirmed by detecting the PCR products for heterogenous double-stranded DNA using T7 endonuclease I (T7EI; New England Biolabs, Ipswich, MA, USA) assay (**Supplementary Figure 2**). These results indicate that direct sequencing might miss minor alleles. Direct sequencing of T_0_ plants demonstrated that the editing efficiency did not differ significantly between *RsGL1a* and *RsGL1b* (75% vs. 62.5%), recognized by the same target sequence to induce genome editing. “The wild-type RsGL1a was not detected in any plant upon the analysis of the cloned sequences of individual alleles; however, the wild-type RsGL1b was detected in plants #3, #4, and #6.”This result showed that the ability of CRISPR/Cas9 to edit wild-type alleles differs even when targeting the same target sequences of paralogous genes. Off-target analysis on eight transgenic plants did not detect off-target mutations by direct sequencing (**Supplementary Figure 3**).

### Analysis of T_1_ generation plants with double mutation of *RsGL1a* and *RsGL1b*

The self-pollinated seeds from four T_0_ plants with double mutations (one with <10 self-pollinated seeds were not used) were collected for phenotypic evaluation. The leaf trichome formation in the T_1_ generation plants was observed visually, and the numbers of hairless and hairy plants were counted (**Table 3**). Hairless phenotypes were obtained in the progenies of all four T_0_ plants, wherein the hairy phenotype was not observed in plants #4 and #5. T-DNA negatives (null-segregant) were obtained for each T_1_ plant (**Supplementary Table 2**), and T_1_ plant #5 was both hairless and T-DNA negative. Visual and microscopic observation revealed considerable differences in trichome formation between hairless and hairy plants. The trichome density on the adaxial and abaxial surfaces of the leaves also showed marked differences (**Figure 2**). Two hairless and hairy progenies of each T_1_ plant (except for #5) were randomly selected. Allele analysis of *RsGL1a* and *RsGL1b* in hairless and hairy phenotypes of T_1_ generation plants is shown in **Tables 4A**, **B**. In hairless plants, all *RsGL1a* and *RsGL1b* were mutated, causing frameshifts, and there were no functional *RsGL1* genes. In contrast, hairy plants had either *RsGL1a*, *RsGL1b*, or both with functional wild-type allele(s). Among the 14 T_1_ generation plants, 5 plants (#4–6, #4–4, #4–9, #5–1, and #5–2) had the allelic composition of *RsGL1a* and *RsGL1b* as detected in T_0_ plants. The T_1_ progenies of plant #5 inherited both *RsGL1a* and *RsGL1b*, #4 inherited *RsGL1b*, and #6 inherited *RsGL1a* detected in their respective T_0_ plants (**Tables 4A**, **B**). The progenies of T_0_ plant #3 had a significantly lower inheritance of alleles (**Tables 4A**, **B**).

## Discussion

Mutations detected in this study were predominantly short (<3 bp) in/del, which might lead to frameshift mutations affecting the amino acid sequence downstream of the target sequence. In contrast, base substitutions were very few compared to in/del induced by the CRISPR/Cas9 system in radish. Concordant with our findings, several other studies conducted on other dicotyledonous plants, such as *Brassica napus* (Yang et al., 2017), *Arabidopsis* (Feng et al., 2014), and tomato (Zhang et al., 2020), have shown that most mutations induced by the CRISPR/cas9 system are insertion, deletion, or complex type which might cause frameshift mutations.

The majority of the mutant alleles in *RsGL1a* and *RsGL1b* in T_1_ plants were also detected in T_0_ plants (**Tables 2** and **Tables 4A, B**), suggesting that the mutant alleles in T_0_ plants were stably inherited in T_1_ plants. However, the mutant alleles in *RsGL1a* and *RsGL1b* in T_0_ plant #3 were not stably inherited. This result could be attributed to the chimera mutation in T_0_ generation, as observed in some *Brassica* species such as *B. napus* (10–100%, Yang et al., 2017) and *B. campestris* (56%, Xiong et al., 2019) and B. oleracea (Lawrenson et al., 2015), suggesting that the novel editing might have occurred during plant regeneration, which uses hypocotyl or cotyledonary petiole as explants that undergo *Agrobacterium* infection during the transformation (Cao et al., 2006; Dun et al., 2011; Muto et al., 2021). Chimeric mutations in the T_0_ generation might obstruct the prediction of the original alleles to be inherited; however, it may increase the diversity of mutant alleles in the T_1_ generation. In summary, we concluded that the mutation pattern introduced by CRISPR/Cas9 in radish is similar to that in other previously reported *Brassica* plants.

As shown in **Tables 4A, B**, T_1_ plants #3–3 and #3–18 with three or more alleles in *RsGL1a* or *RsGL1b* had the wild-type allele and retained T-DNA. This result suggests that Cas9 was active during the reproduction stage from T_0_ to T_1_ generations and mutated the wild-type allele to generate new alleles in the T_1_ generation. Interestingly, the binary vector used in this study has the promoter::Cas9::terminator and promoter::sgRNA suitable for the floral dip method in *A. thaliana* (Fauser et al., 2014), which might have mutated the wild-type allele in the next generation of propagation as reported in *A. thaliana*.

Li et al. (2013) reported that the expression of *RsGL1a* in radish contributes to trichome formation. In this study, we obtained hairy T_1_ plants in which *RsGL1a* was knocked out and *RsGL1b* was functional (#3–18, #4–4, #4–9, #6–3, and #6–4). These results showed that a single knockout of either *RsGL1a* or *RsGL1b* did not affect trichome formation; however, double knockout plants produced hairless leaves, suggesting that these two alleles contribute to trichome formation independently. Furthermore, the identification of “double knockout” T_1_ plants indicates that neither *RsGL1a* nor *RsGL1b* is essential for the basic growth performance of the plant (**Figure 2F**). Therefore, these plants can be used for breeding hairless radishes. However, further investigations are needed to validate the effects of the “double knockout” on the tolerance to several stresses, yield, and edible quality of radish.

In conclusion, we successfully performed genome editing using the CRISPR/Cas9 system targeting the genes associated with leaf trichome formation in radish. We showed that although the radish genome has two to three redundantparalogs, efficient genome editing can be achieved using a single sgRNA specific for the modification of multiple paralogs simultaneously. This study showed that genome-edited radish without T-DNA (null-segregant) could be obtained. This radish is useful as a novel breeding material because it might not be treated as a transgenic plant. The findings demonstrated that the CRISPR/Cas9 system is a highly efficient tool for genome editing in radish. However, limited varieties of radishes can be genetically transformed (Muto et al., 2021). Moreover, whether the wild-type allele in F_1_ plants can be edited by crossing the mutated ‘Pirabikku’ plants obtained in this study with other radish varieties should be investigated in the future. We believe the CRISPR/Cas9 mediated targeted gene mutagenesis can contribute to the efficient elucidation of gene function mechanisms and assist in the acceleration of molecular breeding of novel radish varieties.

## Data availability statement

The original contributions presented in the study are publicly available. This data can be found here: DDBJ, LC719984 & LC719985.

## Author contributions

NM performed all the experiments, analyzed the data, created the Figures and Tables, and wrote the manuscript. TM conceived the study, participated in its design, and helped to draft the manuscript. All authors have read and approved the final manuscript.

## Funding

This study was supported by grants from the Tokyo NODAI General Director Project of Tokyo University of Agriculture (46406790). This work was also supported by the JST SPRING (grant number JPMJSP2122).

## Acknowledgments

We would like to thank Dr. Masaki Endo and Dr. Seiichi Toki from NARO, Japan for providing the vectors pZKAtU6gRNA_FFCas9_NPTII and pUC19_AtU6_ccdB_oligo, respectively. We would like to thank Editage (www.editage.com) for English language editing.

## Conflict of interest

The authors declare that the research was conducted in the absence of any commercial or financial relationships that could be construed as a potential conflict of interest.

## Publisher’s note

All claims expressed in this article are solely those of the authors and do not necessarily represent those of their affiliated organizations, or those of the publisher, the editors and the reviewers. Any product that may be evaluated in this article, or claim that may be made by its manufacturer, is not guaranteed or endorsed by the publisher.
