# Supplementary material for: CRISPR/Cas9-mediated genome editing of RsGL1a and RsGL1b in radish (Raphanus sativus L.)
Source: Front Plant Sci. 2022 Oct 13;13:951660. doi: 10.3389/fpls.2022.951660 (PMC9606758; doi:10.3389/fpls.2022.951660)
Supplement: Supplementary file 1 [file DataSheet_1.docx]

***Supplementary Material***

Supplementary Figures and Tables

Supplementary Figure 1. Nucleotide and protein sequences of RsGL1a and RsGL1b in radish cv. ‘Pirabikku’.

Supplementary Figure 2. Detection of DNA mismatches by T7 Endonuclease I

Supplementary Figure 3. Detection of mutations in the off-target region of the orthologue of *AtMYB23*.

Supplementary Table 1. Sequences of primers used in this study.

Supplementary Table 2. Confirmation of T-DNA insertion in T_1_ plants

## Supplementary Figures

>Pirabikku_*RsGL1a*_Nucleotide

ATGAGAATGAGAAGGAGATCAGAGGAACGAGAAAATCATCAG**GAATACAAGAAAGGGTTATGG**ACAGTTGAAGAAGACAACATTCTTATAGATTATGTCCAAGCTCACGGCACAGGCCTATGGAATCGCATTGTCAGGAAAACTGGTCTCTCTCTCTCTCTCTCCATTTATAAATTTTACCGATCTCTAGCTAGAACATACATGTATATGTAACTACTTGATTTGGTATTAATTTTAGGGTTGAAGAGGTGTGGAAAAAGCTGTAGACTGAGATGGATAAATTATTTGAGCCCTACTGTGAATAAAGGCAATTTCACTGAGCAAGAAGAAGACCTCATTATTCGTCTCCACAAACTCCTCGGCAACAGGTACGTCTACTGAGCTCCCTTTCTATAAATTTTACATTATATTATATAACATGTCTTCACTTATAGGCTTCAACTCTTAAACATAAAGACTTGCAAAATTTCTTTATTGTTTTTGTATCGAACATTCGAACTTGAACATTCGAACTTAACTGATGTAACGTTTTAATTTTAATTAATGTTATTCCTAAAAAGTTTTGTGATGTTTTTGTCAAAAAAAATTTCAAATTTTTAATATGTTCAAAAATATCTCCATTTGTAACCTTTTTTGTAAACTTCCATTTGTAACCTCTTTCACACTATTTAATTATTTATGAATGATTTACAAATGCATTACTCATTTATGAATGATTTACAAATGTCAGCAAAATCATACACATCTACATATATGAATTATATAGGTTTATATAATTAAATTTATAGAATGTGATTTTATCATCTGAAGTATGATATTTTCTTTCCAATCCAGGTGGTCTTTGATAGCTAAAAGAGTACCTGGACGAACAGATAACCAAGTCAAGAATCACTGGAACACTCATCTCAGTAAGAAGTTCGTCGGAGATTATTCCTCCGCAGTCAAAACCACCGGAGAAGACAACTCTCCAGCGTCACTGCTCATTTCCGCCGCCACAGCTTCTAATCGTCAACACCAACAAGACAAAATCTGCGCCAAGAGCTTCGACGGCCTCGTACCAGCTTCGTACGAGAAATTGACACATAGCGACGTCGTATTGGGAAATACTAATCATCCAAGTCTCGACTTCAAAGAAAGGAACAACTTTGATGGCAGTAACGCATTCTGGTTTAATGAAGACGACTTCGAGCTCGTGAGTTCATTCGCTATGATGGATTTTGCTTCGAGTGATATTGGCTACTACCTCTAG

>Pirabikku_RsGL1a_Protein

MRMRRRSEERENHQEYKKGLWTVEEDNILIDYVQAHGTGLWNRIVRKTGLKRCGKSCRLRWINYLSPTVNKGNFTEQEEDLIIRLHKLLGNRWSLIAKRVPGRTDNQVKNHWNTHLSKKFVGDYSSAVKTTGEDNSPASLLISAATASNRQHQQDKICAKSFDGLVPASYEKLTHSDVVLGNTNHPSLDFKERNNFDGSNAFWFNEDDFELVSSFAMMDFASSDIGYYL

>Pirabikku_*RsGL1b*_Nucleotide

ATGAGAAGGACGAGAAGAACAGATGAAGGAGAGAATCATCAA**GAATACAAGAAAGGGTTATGG**ACAGCAGAAGAGGACAACATTCTTAGAGATTATGTCCTTACTCACGGCAAAGGCCAATGGAACCGCATCGTCAGGAAAACTGGTCTCTCTTTCTTTGTGAACATATATATATATATATATATATATATATCACTAGCTAGATCATATATATACATATTCTCAATTTGATTTGGTTTACTTTATATTTATTTAGGGCTGAAGAGGTGTGGAAAAAGCTGTAGACTTAGATGGATAAATTATCTGAGCCCTAATGTGAATAAAGGCAATTTCACTGAACAAGAAGAAGACCTCATTATTCGTCTCCACAAGCTCCTCGGCAACAGGTACCTCTCTCTCTCTCTCTCTCTCTCTAAAAAACATGTCTTCATATTTACAAGCTTCAATTTTTAGACCAGTGAACTGCAAATGGTGTGCAAACATAAAAATAAAGACTTACAAAAGAATTTTTTTATTGTTTCTTCAATTAAACTTAACTAATGTGTTGAGATATATATTTTATCAATTTTATTGCTGTTTCTTCTAACTGTTTTAAGTGTTTTTTAGTCAGAAAACAAAATAATGTTACTTTTAAAAATGTTCAAAATTCTGTAAATATCTTCATCTATTCTCTTTCTCTCACAGTGTAGGTACATAAATATGAATATACATCATTTATGAAGATTATACAAATTAATGTCAAGGGAAACAATCATAGACATCTACTGAGATCCATAACTGTACATCTACAAGAATTATATTATATAATGGTCGGTTCATGTTATATATAGTATAGTATGTTTTTATGATCTTATTATGATATATTCTCTCCCAAATCCAGATGGTCTTTAATAGCTAAAAGGGTGCCTGGAAGGACAGATAACCAAGTCAAGAATCATTGGAACACTCATCTCAGCAAAAAAATCGTAGGGGATTATTCCTCCGCTGTCAAAACCACCGGAGAAGAAAATTATCCACCGTCACTACTCATCACCGGCGCAACAGCTTCTTGTCATCATCAACAAGACAAAATCTGTGACAAGAGCTTCGAGGGCCTAGTATCAGCTTATTACGAGAATAAACCAAAAGCAGGGTTGACACAGAGAGAAGTCATGGTGGAAAACACTAATGATTCGAGTATTTACTTCAAAGAGAGGAACAACTTTGATAGCAGTAACGCTTTCTGGTTTAATGAAGACGATTTTGAGATGAATTCATTTGTTATGATGGATTTTGCGTCGGGTGATATTGGCTACTGCCTCTAGTTATGA

>Pirabikku_RsGL1b_Protein

MRRTRRTDEGENHQEYKKGLWTAEEDNILRDYVLTHGKGQWNRIVRKTGLKRCGKSCRLRWINYLSPNVNKGNFTEQEEDLIIRLHKLLGNRWSLIAKRVPGRTDNQVKNHWNTHLSKKIVGDYSSAVKTTGEENYPPSLLITGATASCHHQQDKICDKSFEGLVSAYYENKPKAGLTQREVMVENTNDSSIYFKERNNFDSSNAFWFNEDDFEMNSFVMMDFASGDIGYCL

**Supplementary Figure 1.** Nucleotide and protein sequences of *RsGL1a* and *RsGL1b* in radish cv. ‘Pirabikku’. Target sequence and PAM (21-mer) are shown in bold font. DNA-binding domains of RsGL1a and RsGL1b are shown with gray background.

**
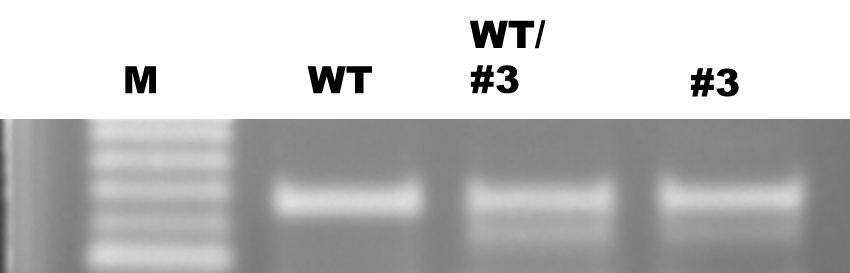
**

**Supplementary Figure 2.** Detection of DNA mismatches by T7 Endonuclease I. M, Molecular weight marker; WT, PCR product of *RsGL1a* in wild-type; #3, PCR product of *RsGL1a* in T_0_ plant #3.

PAM

PAM

PAM


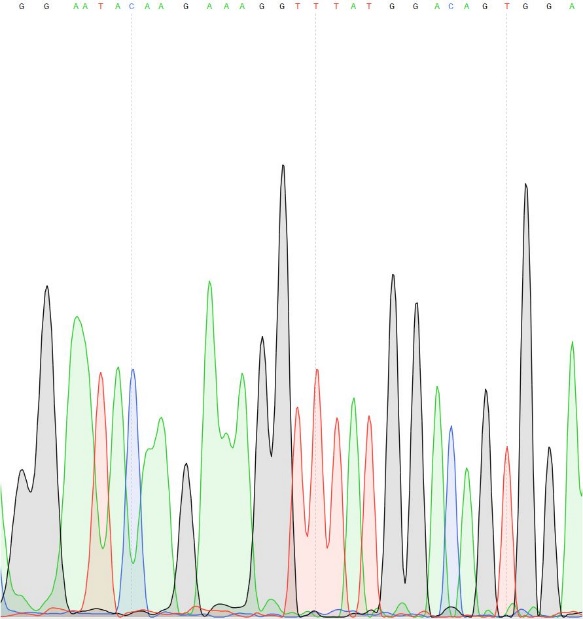


**#3**


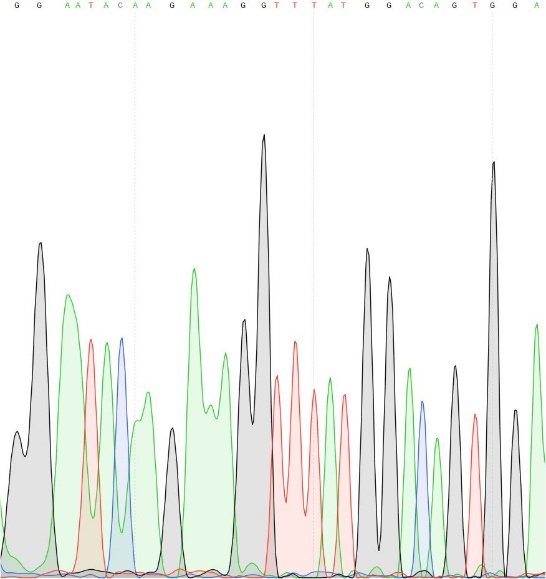

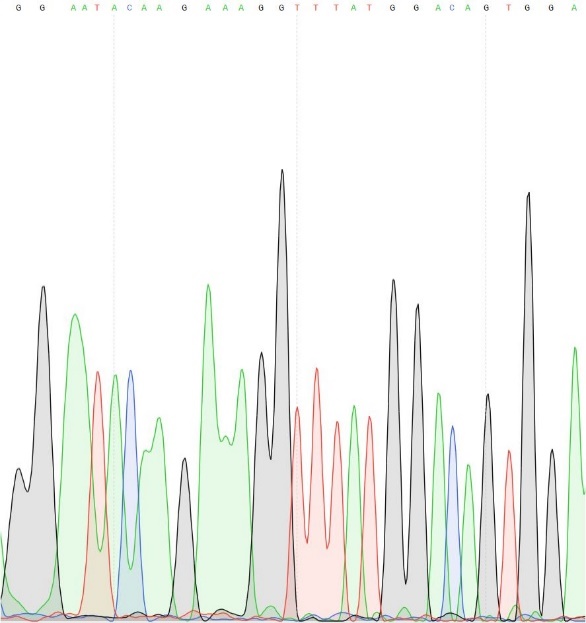

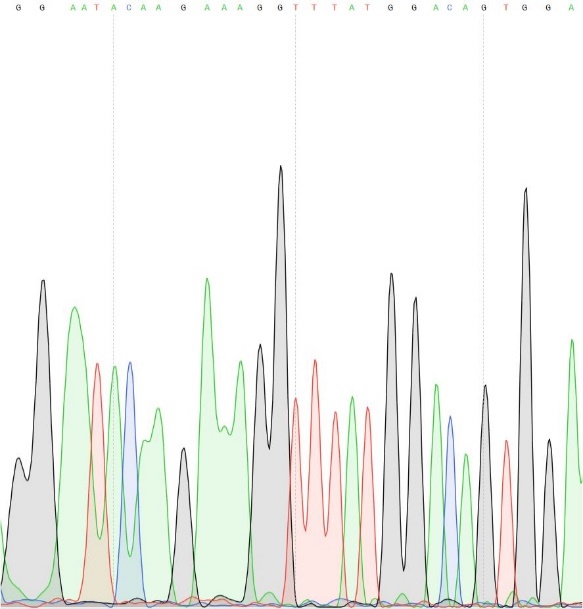


**#2**


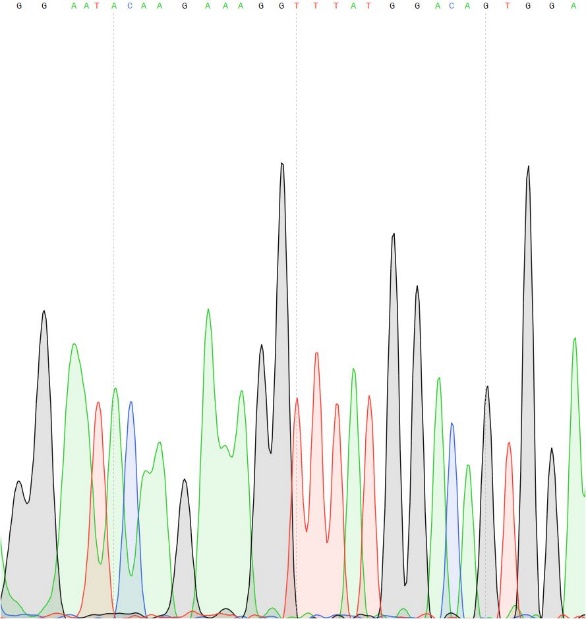

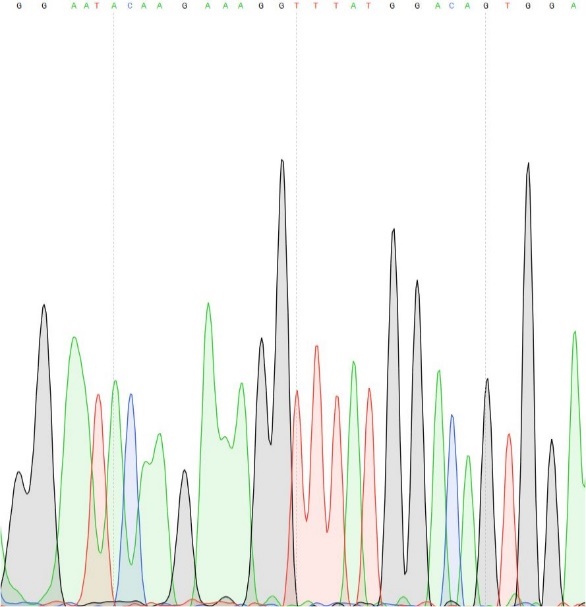


**#1**

**#8**


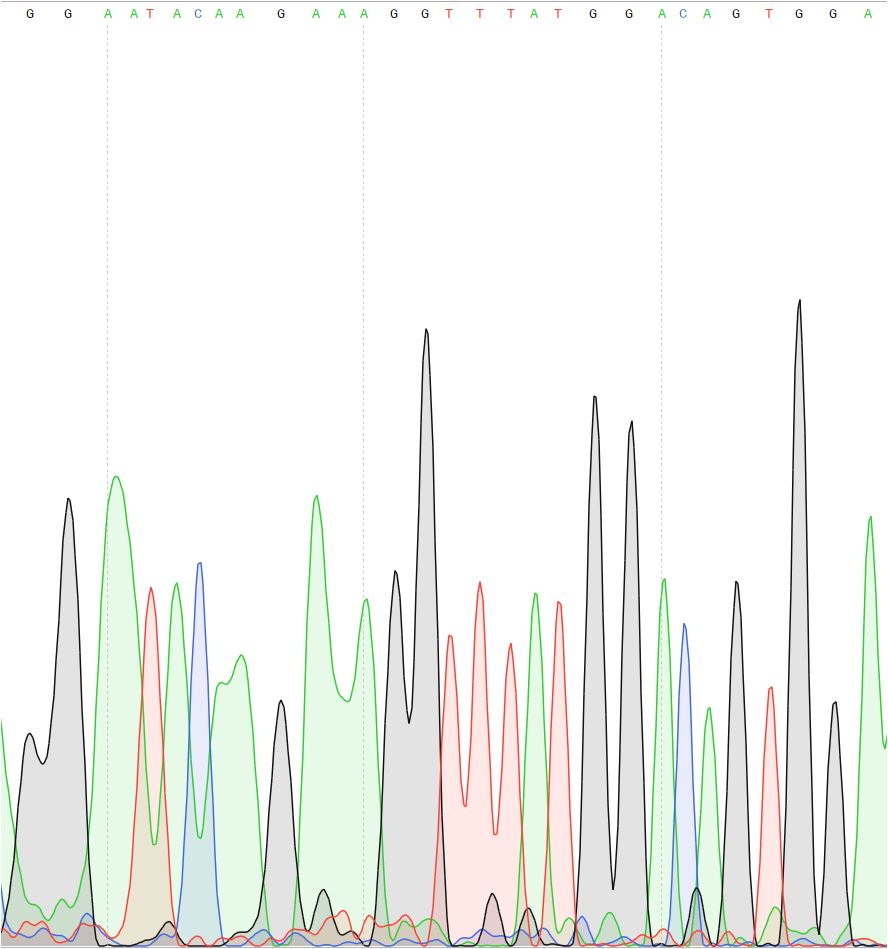

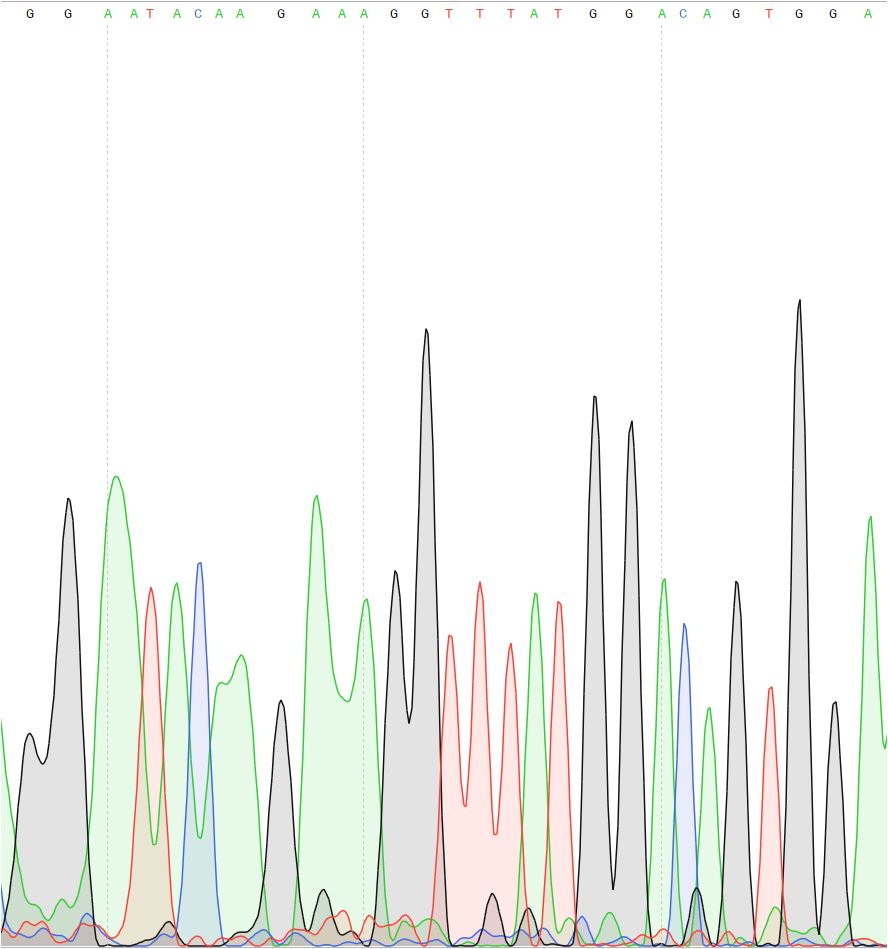


**#7**


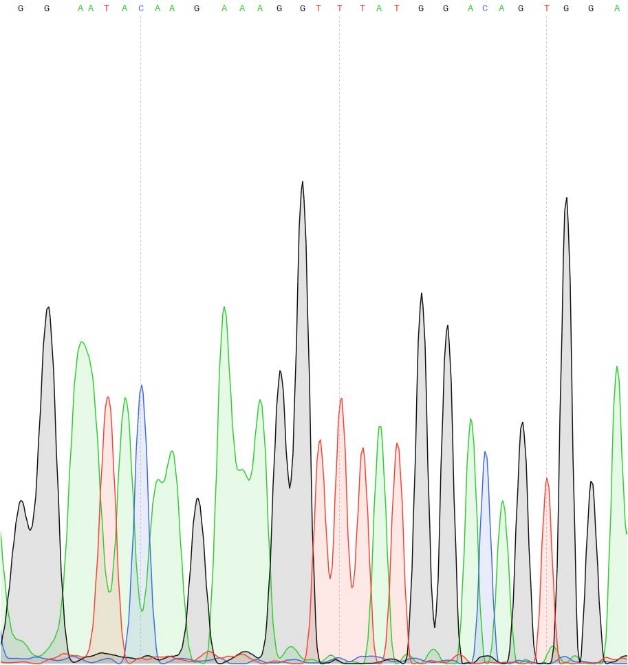

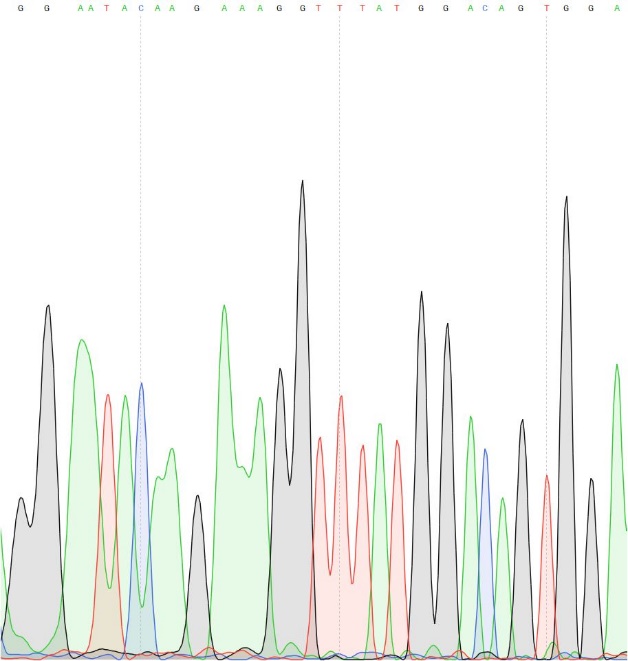

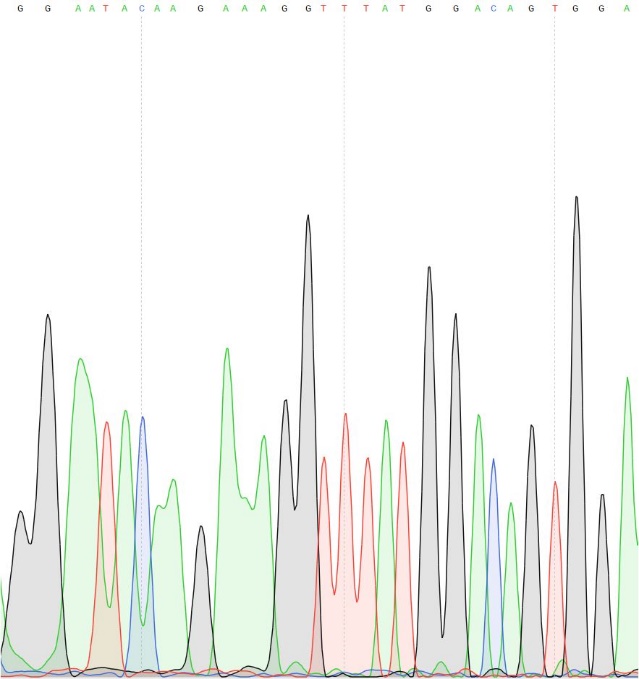

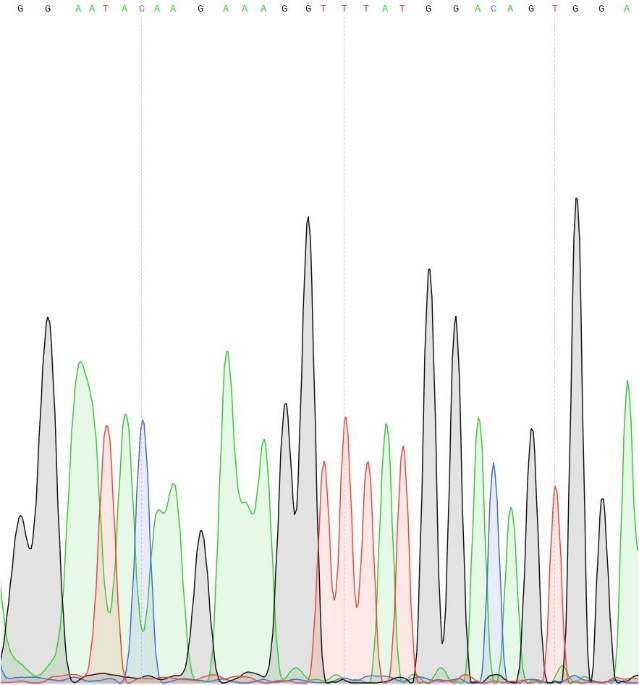


**#6**


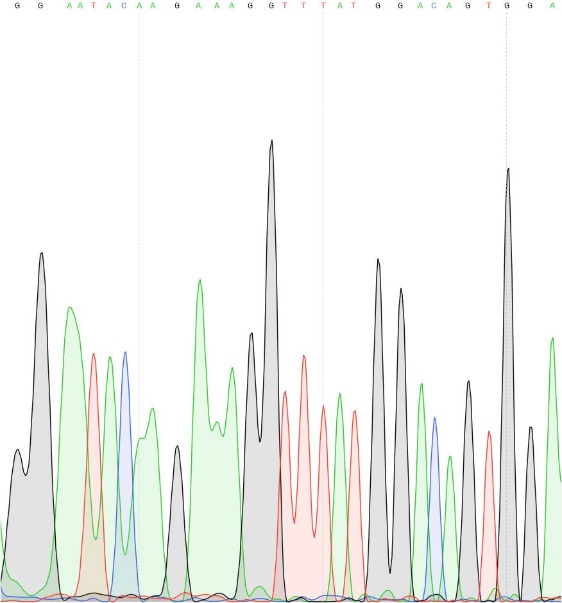


**#5**


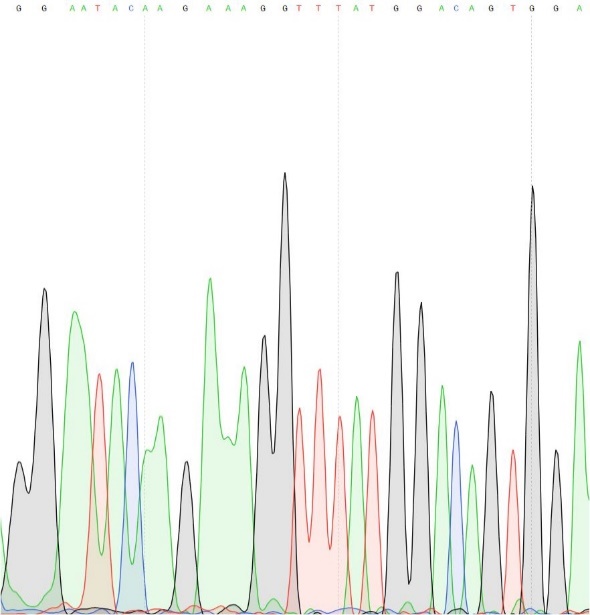

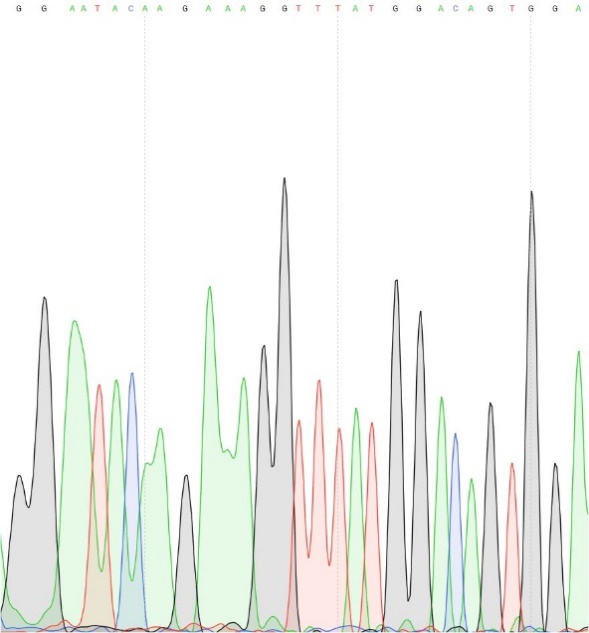

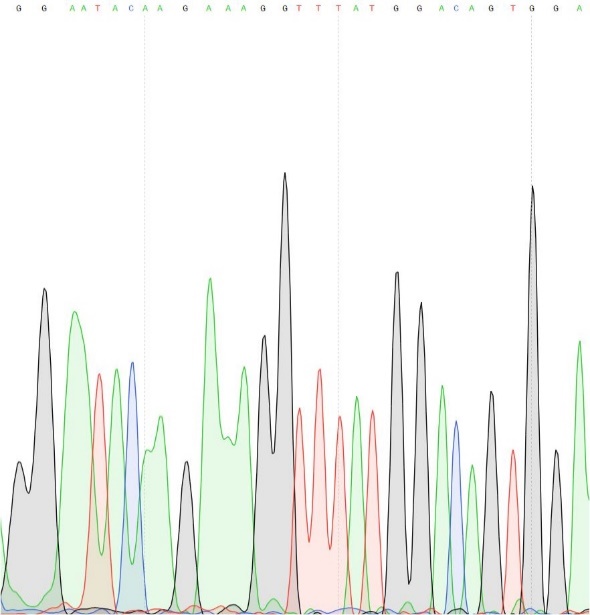


**#4**

PAM

PAM

PAM

PAM

PAM

**Supplementary Figure 3.** Detection of mutations in the off-target region of the orthologue of AtMYB23. The number in each figure indicates the name of the T_0_ plants. The red dotted line indicates the upstream 2-3 bases of PAM.

**2. Supplementary tables**

| **Supplementary Table 1.** Sequences of primers used in this study. | | |  |  |
| --- | --- | --- | --- | --- |
| Primer name | Sequence (5’-3) | Purpose |  |  |
| RsGL1a_F1 | ATGAGAATGAGAAGGAGATCAGAGG | Sequencing of RsGL1a | | |
| RsGL1a_R1 | GGAGTTTGTGGAGACGAATAATGAGG |  |  |  |
| RsGL1a_F2 | CCTCATTATTCGTCTCCACAAACTCC |  |  |  |
| RsGL1a_R2 | CCAGGTACTCTTTTAGCTATCAAAGACCAC |  |  |  |
| RsGL1a_F3 | GCTAAAAGAGTACCTGGACGAACAGATAAC |  |  |  |
| RsGL1a_R3 | CTAGAGGTAGTAGCCAATATCACTCGAAGC |  |  |  |
| RsGL1b_F1 | ATGAGAAGGACGAGAAGAACAGATGAAGG | Sequencing of RsGL1b | | |
| RsGL1b_R1 | TACAGCTTTTTCCACACCTCTTCAG |  |  |  |
| RsGL1b_F2 | GGCTGAAGAGGTGTGGAAAAAGCTG |  |  |  |
| RsGL1b_R2 | TTTGCTGAGATGAGTGTTCCAATG |  |  |  |
| RsGL1b_F3 | AATAGCTAAAAGGGTGCCTGGAAGG |  |  |  |
| RsGL1b_R3 | CATAACTAGAGGCAGTAGCCAATATCACC |  |  |  |
| RsGL1a_PCR_F | AAGGAGATCAGAGGAACGAGAAAAT | Mutation detection | |  |
| RsGL1a_PCR_R | CTTGCTCAGTGAAGTTGCCTTTATT |  |  |  |
| RsGL1b_PCR_F | ACGAGAAGAACAGATGAAGGAGAGA |  |  |  |
| RsGL1b_PCR_R | TTCAGTGAAATTGCCTTTATTCACA |  |  |  |
| RsGL1a_SEQ | CCAGTTTTCCTGACAATG |  |  |  |
| RsGL1b_SEQ | CACAAAGAAAGAGAGACCA |  |  |  |
| Hpt_F | GATATGTCCTGCGGGTAAATAGCTG | Confirmation of transformation | | |
| Hpt_R | GTAGTGTATTGACCGATTCCTTGCG |  |  |  |
| orf687_F | CCGGATCTTGTGATTTCTCTC | Detection of fertile plants | | |
| orf687_R | GCCTACATGTCGTTTCAAAC |  |  |  |
| RsMYB23_F | TGAATTATCCGCCCTCTCTC | Sequencing of RsMYB23 | |  |
| RsMYB23_R | GACCTGTCTTCTTGGCGATG |  |  |  |
| RsMYB23_SEQ | AAGAACAGTAAAAAGATGAG | Off-target detection | |  |

| **Supplementary Table 2.** Confirmation of T-DNA insertion in T_1_ plants | | | |
| --- | --- | --- | --- |
| T_0_ plant No. originated | T-DNA | | Total |
|  | Positive | Negative |  |
| #3 | 16 | 3 | 19 |
| #4 | 10 | 4 | 14 |
| #5 | 14 | 3 | 17 |
| #6 | 7 | 2 | 9 |
